# Local and systemic immune responses induced by intranasal immunization with biomineralized foot-and-mouth disease virus-like particles

**DOI:** 10.3389/fmicb.2023.1112641

**Published:** 2023-02-03

**Authors:** Shuo Li, Ruichong Zhao, Hetao Song, Songjia Pan, Yun Zhang, Hu Dong, Manyuan Bai, Shiqi Sun, Huichen Guo, Shuanghui Yin

**Affiliations:** ^1^State Key Laboratory of Veterinary Etiological Biology, College of Veterinary Medicine, Lanzhou University, Lanzhou Veterinary Research Institute, Chinese Academy of Agricultural Sciences, Lanzhou, Gansu, China; ^2^College of Veterinary Medicine, Gansu Agricultural University, Lanzhou, China; ^3^Yunnan Tropical and Subtropical Animal Virus Diseases Laboratory, Yunnan Animal Science and Veterinary Institute, Kunming, Yunnan, China

**Keywords:** foot-and-mouth disease virus, biomineralization, virus-like particles, mucosal immunity, adjuvant

## Abstract

**Introduction:**

Foot-and-mouth disease virus (FMDV) infects the host by invading mucosal epithelial cells of the respiratory or digestive tract. Therefore, establishing a specific antiviral mucosal immune barrier can effectively block viral invasion.

**Methods:**

We evaluated local mucosal and systemic immune responses elicited by intranasal immunization of mice with foot-and-mouth disease (FMD) calcium phosphate mineralized virus-like particles (CaP-VLPs) and tested whether three commercial mucosal adjuvants enhanced the immunogenicity of the antigen. The biosafety of the vaccine was verified through gross observation and pathological analysis of the lungs.

**Results:**

CaP-VLPs effectively induced secretion of IgA (sIgA) from multiple sites in mouse mucosa and produced anti-FMD-specific IgG in the serum. Splenic lymphocytes specifically proliferated and secreted IFN-γ following antigen stimulation, indicating the vaccine can induce a certain level of cellular immune response. Finally, the pathological examination confirmed that CaP-VLPs did not cause substantial damage to the lungs of animals after immunization *via* mucosal administration. Notably, the vaccine mixed with S adjuvant increased the content of sIgA and serum IgG, and the high level of IgG in serum was maintained at least 7 weeks.

**Discussion:**

Overall, this study reveals that FMD CaP-VLPs can induce good local mucosal immune and systemic immune response through intranasal immunization, and the immune response was specifically enhanced by S adjuvant. These data support that CaP-VLPs-S as a candidate mucosal vaccine for the prevention of FMD vaccine infection.

## Introduction

Foot-and-mouth disease (FMD) is a severe infectious disease caused by foot-and-mouth disease virus (FMDV) and is characterized by blisters and ulcers on the oral epithelium and hoof skin of cloven-hoofed animals such as cattle, sheep, and pigs as the main clinical symptoms (Grubman and Baxt, 2004). FMD is still prevalent in many developing countries and has caused huge economic losses (Knight-Jones and Rushton, 2013; Stenfeldt et al., 2014). Intramuscular vaccination by injection is the most economical and effective measure to prevent and control the spread of FMD outbreaks (Parida, 2009). However, the FMDV can spread in the air in aerosol form, and the protection provided by traditional vaccines does little to stop the virus from invading (Arzt et al., 2010; Pacheco et al., 2010). Mucosal vaccines can induce both mucosal site responses and systemic immune responses (Lycke, 2012), providing better protection for animals. Hence, investigations are ongoing to find a good mucosal immunity vaccine to better prevent and control FMD.

Virus-like particles (VLPs) vaccines are empty capsid particles assembled from viral capsid proteins (Fuenmayor et al., 2017). Several studies have shown that VLPs vaccines are well suited for mucosal immunity, and demonstrate excellent immune efficacy and good safety (Revaz et al., 2007; Serradell et al., 2019; Rothen et al., 2022). The FMD VLPs vaccine produced by our laboratory has a good immune effect (Guo et al., 2013); we also introduced a calcium phosphate shell into the vaccine using biological mineralization technology, which greatly enhanced the heat resistance of the vaccine and optimized the conditions of vaccine use (Du et al., 2019; Guo et al., 2021). Interestingly, complexes such as CaP-antigens can effectively induce mucosal immune responses (He et al., 2002; Knuschke et al., 2013), and the CaP shell can effectively shield the epitope of the vaccine and overcome the immune interference caused by multiple immunizations (Wang et al., 2016). These bring reliable technical support for FMD CaP-VLPs for intranasal immunization.

Encouraged by these results, we report the potential use of FMD CaP-VLPs as a mucosal vaccine for FMD and perform a simple screening of suitable mucosal adjuvants for CaP-VLPs. The results showed that intranasal immunization of mice with CaP-VLPs vaccine adjuvanted with S could induce a potent specific mucosal and systemic immune response against FMDV, providing comprehensive protection for animals. Our results will provide new ideas for the development and application of FMD mucosal vaccines.

## Materials and methods

### Preparation and characterization of FMD CaP-VLPs

FMD structural protein expressed in *Escherichia coli* was purified by nickel ion affinity chromatography, and assembled into FMD VLPs *in vitro* after tag removal (Guo et al., 2013). The target protein was detected by SDS-PAGE and Western blot analysis.

The biomineralization of FMD VLPs was completed by a well-established method in the laboratory (Du et al., 2019) and the morphology of CaP-VLPs was examined by transmission electron microscopy (TEM; HT7700; Hitachi, Tokyo, Japan). CaP-VLPs solution was dropped onto a carbon-coated 300-mesh copper grid. The excess sample solution was removed with filter paper and dried at room temperature prior to observation. For VLPs, it is necessary to drop the phosphotungstic acid solution for negative staining for 30 s after the VLPs solution has dried; excess solution was removed with filter paper before observation. The particle size of VLPs and CaP-VLPs was measured by dynamic light scattering using a Zetasizer Nano (Malvern Zetasize Nano ZS90; Worcestershire, UK).

### Adjuvants and immunization

In this study, MONTANIDE™ GEL 02 PR (Number: 36084X) and MONTANIDE™ IMS 1313 VG NST (Number: 36071H; SEPPIC company), and STR products (patent number: ZL201310018498.X, China Biotech Co. Ltd.) were selected as adjuvants to mix with FMD CaP-VLPs. The above adjuvant products are abbreviated as G, I, and S, respectively.

All experimental procedures involving BALB/c mice complied with the Animal Welfare Procedures and Guidelines of the People’s Republic of China for Animal Ethics. Experiments were approved by the Animal Ethics Committee of Lanzhou Veterinary Research Institute, Chinese Academy of Agricultural Sciences. BALB/c female mice (6–8 weeks old, 16–20 g) were randomly divided into five groups (20 mice in each group). On days 0, 7, and 14, mice in the experimental group were intranasally immunized with 50 μg CaP-VLPs each time, and mice in the control group were immunized with phosphate-buffered saline (PBS). Serum and mucosal samples were collected at different time points after immunization for antibody analysis. Lung lavage fluid, small bowel lavage fluid, and fecal pellets were collected from mice at different time points after immunization and immersed in ice-cold buffer overnight. The buffer was a PBS solution containing soybean trypsin inhibitor (Gibco; 0.1 mg/ml), EDTA (50 mM), Pefabloc (Merk; 0.35 mg/ml), bovine serum albumin (BSA; 0.1 mg/ml). The buffer formula is from Christensen’s work (Christensen et al., 2019). After incubation, lung and small bowel lavage fluid, and fecal pellet suspensions were clarified and recollected by centrifugation at 12000 g for 30 min at 4°C. The supernatants were frozen and analyzed at a later date by the indirect enzyme-linked immunosorbent assay (ELISA).

### ELISA assays

Indirect ELISA: Inactivated type-O FMDV from the FMD type-O liquid-phase blocking ELISA (LPBE) antibody detection kit was used as coating antigen on ELISA plates (Costar), and the free binding sites were blocked with 5% skim milk in PBS-Tween-20 (PBST). Mucosal samples to be tested and goat anti-mouse IgA (HRP; Abcam) were added in sequence. TMB was used as substrate to detect antibody responses. After the reaction was stopped, the optical density (OD) was measured at 450 nm. The contents of different types of IgG in serum at 28 days after immunization were detected by indirect ELISA. The ELISA plate was coated with inactivated type-O FMDV overnight at 4°C, blocked with 5% skim milk in PBST, and serum after immunization for 28 days (diluted 100 times) and goat anti-mouse IgG1, IgG2a (HRP; Abcam) were added. TMB was used as substrate and the plate was read at 450 nm.

Liquid phase blocking ELISA: Orbital venous blood of immunized mice was collected and the serum was isolated by centrifugation. Specific antibodies in the serum of immunized mice were detected and analyzed using the FMD type-O LPBE antibody detection kit (Lanzhou Shouyan Biotechnology Co., Ltd.).

Double sandwich ELISA: Serum interferon (IFN)-γ and interleukin (IL)-4 on day 28 after immunization were detected by mouse IFN-γ and IL-4 ELISA kits (Shenzhen Xin Bosheng Biological Technology Co., Ltd.).

### Detection of neutralizing antibodies

Serum of mice immunized for 28 days was inactivated at 56°C for 30 min, dilute the serum to be tested with cell culture medium containing 2% fetal bovine serum (FBS) (v/v) (inactivated) and set negative serum control and positive serum control. The FMDV strain O/China99 was diluted to 200 TCID_50_/0.1 ml, and the same volume was mixed with the diluted serum and incubate at 37°C for 1 h. Each well was seeded with 1 × 10^6^ BHK-21 cells and incubated for 72 h in a temperature chamber containing 5% CO_2_ at 37°C. Cytopathic effects were then observed under an inverted microscope. The serum dilution that protected 50% of cells against cytopathic changes was calculated by the Reed-Muench method.

### T-lymphocyte proliferation assays

On day 28 after immunization, mice were sacrificed after anesthesia with ether and spleen lymphocytes were aseptically isolated from mice. Cells were resuspended in RPMI-1640 containing 10% FBS (v/v) and 1% penicillin–streptomycin solution (v/v). The cells added to a 96-well cell culture plate (Corning, 10^6^ cells/well, 100 μl), 1 μl Concanavalin A (Con A) (Sigma, 1 mg/ml), and type-O FMD VLPs (1 mg/ml) were added to the wells as positive control and a blank control group containing only culture medium but not cells was established. After 72 h of culture, the culture supernatants before and after the stimulation of lymphocytes in each group were analyzed for IFN-γ content. The OD at 490 nm was detected, and finally, the stimulation index (SI) was calculated as follows:


$$SI=\frac{\begin{array}{l} OD490\;\mathrm{of stimulation group}-\\ OD490\;\mathrm{of}\;\mathrm{blank control group} \end{array}}{\begin{array}{l} OD490\;\mathrm{of negative control group}-\\ OD490\;\mathrm{of}\;\mathrm{blank control group} \end{array}}$$

### Measurement of lung weight and lung index

Body weight changes of the mice in each group after immunization were detected. On day 28, mouse lungs were aseptically removed, observed, photographed, and weighed. The lung index was calculated as follows:


$$\mathrm{Lung index}=\frac{\mathrm{Lung weight}\;\left(g\right)}{\mathrm{Body weight}\;\left(g\right)}\times 100\%$$

### Histopathological examination

Lung tissues were removed and fixed in 4% paraformaldehyde, dehydrated in an alcohol gradient, transparent with toluene, embedded in paraffin, sectioned, and stained with hematoxylin and eosin (H&E) and Masson trichrome.

H&E: Sections were dewaxed to water, stained with hematoxylin for 10 min, differentiated with 1% hydrochloric acid alcohol for several seconds, stained with eosin for 5 min, dehydrated with gradient alcohol, transparent with toluene, and sealed with neutral gum. Histopathological changes were observed, recorded, photographed with a microscope (BA210Digital, USA), and lung inflammation was scored (National Toxicology Program, 2000, 2007).

Masson trichrome: Sections were dewaxed to water, treated with Bouin’s solution at 37°C for 2 h, stained with celestine blue droplets for 2–3 min, stained with Mayer’s hematoxylin for 3 min, differentiated with acidic ethanol for several seconds, and stained with Ponceau Fuchsin staining solution for 10 min. Phosphorous molybdic acid solution was used for color separation for 10 min followed by aniline blue staining solution for 5 min, weak acid solution for 2 min, rapid dehydration with 95% alcohol, dehydration with anhydrous ethanol, clear xylene, and sealing with neutral gum. The distribution and composition of lung fibers were observed; fibrils, mucus, and cartilage were blue, muscle fibers, cellulose, muscle, glia, and cytoplasm were red, red blood cells were orange-red, and nuclei were dark-blue. Slices were imaged under a microscope (BA210Digital, USA) at 400× magnifications. Three fields of view were randomly selected and Image-Pro Plus (Media Cybernetics, USA) was used to measure the fibrous tissue area in the collected images. Fibrous tissue was calculated as follows (de Castro et al., 2017):


$$\mathrm{Percentage of expression area}=\frac{\mathrm{fibrous tissue area}}{\mathrm{field of view area}\;\left(\mathrm{pixel area}\right)}\times 100\%$$

### Statistical analysis

GraphPad Prism 8.0 was used for graphing the data in each group. All statistical analyses were conducted with one-way analysis of variance (ANOVA) by SPSS 22.0. *p* < 0.05 (*) were considered statistically significant, *p* < 0.01 (**) were considered highly significant.

## Results

### Characterization of the CaP-VLPs complex

**Figure 1:**
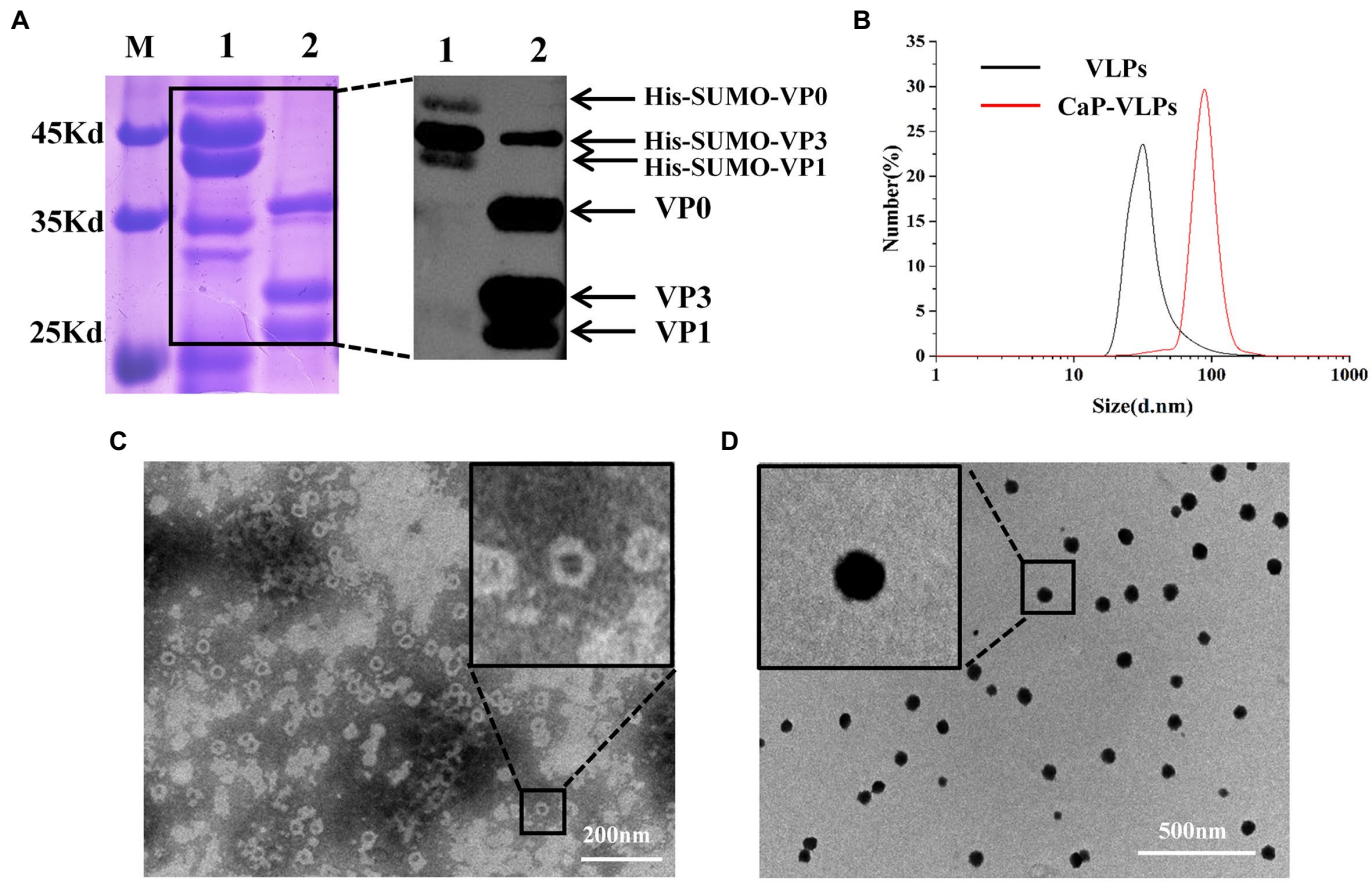
Characterization of mineralized FMD CaP-VLPs. **(A)** SDS-PAGE and Western blot analysis of FMD VLPs. Lane 1: FMD structural proteins with His-SUMO tag, lane 2: FMD VLPs. **(B)** Identification of FMD VLPs and FMD CaP-VLPs with dynamic light scattering (DLS). **(C)** Transmission electron microscopy (TEM) observation of FMD VLPs. **(D)** TEM observation of FMD CaP-VLPs.

The FMD VLPs were assembled from three full-length foot-and-mouth disease structural proteins VP0 (33 kDa), VP3 (24 kDa) and VP1 (23 kDa) *in vitro*. SDS-PAGE and Western blot analysis (Figure 1A) showed that the apparent molecular weight of the three FMD structural proteins was consistent with the expected size. Hydration diameters of the two complexes were measured by dynamic light scattering (DLS). As shown in Figure 1B, the hydrodynamic diameter of VLPs was approximately 37 nm, while the diameter of CaP-VLPs was 92 nm. The morphology of VLPs and CaP-VLPs was observed using TEM, the ultrastructure of CaP-VLPs showed a dense nanoparticle structure. The particle size of CaP-VLPs was significantly higher than that of VLPs (Figures 1C,D), which is consistent with the results observed by DLS, indicating that calcium and phosphorus elements successfully encapsulated VLPs. The above results confirm the successful acquisition of VLPs and CaP-VLPs.

### Local mucosal responses

**Figure 2:**
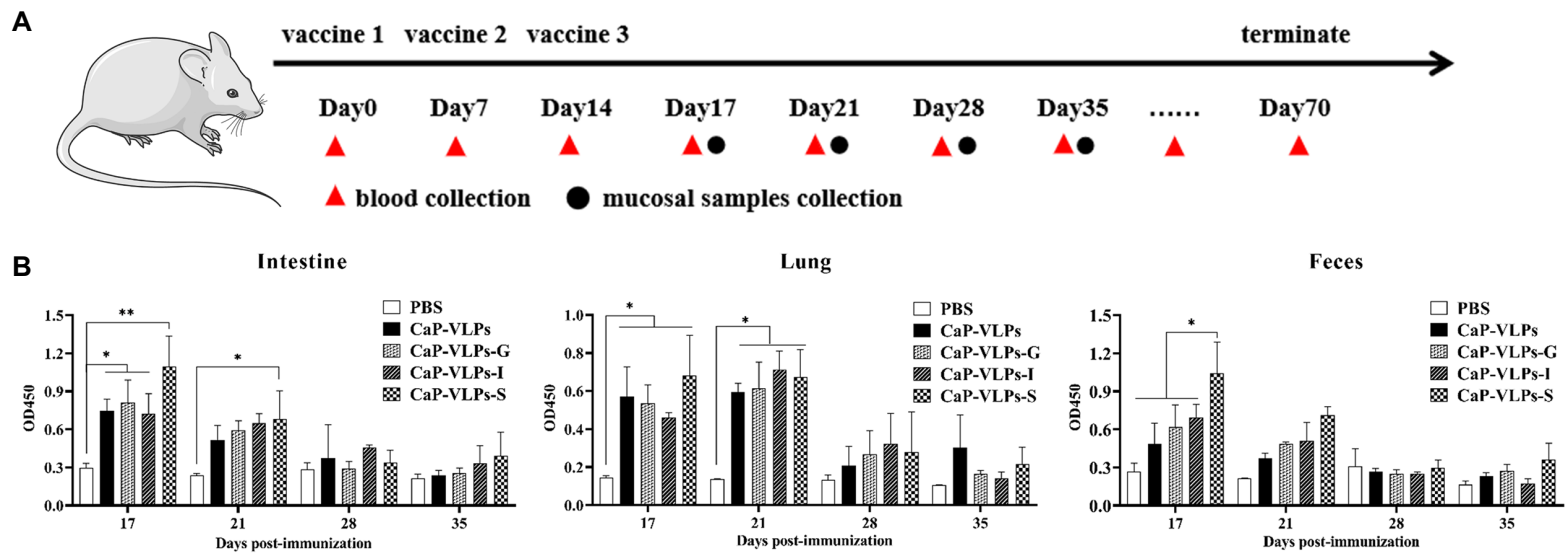
Immune responses of regional mucosal sites elicited by CaP-VLPs. **(A)** Overview of the immunization schedule. **(B)** BALB/c mice (*n* = 20/group) were immunized with CaP-VLPs with or without adjuvant three times on days 0, 7, and 14. IgA in the intestines, lung, and feces of mice was monitored by indirect ELISA. Asterisks indicate significant differences (*p < 0.05, **p < 0.01).

To determine whether intranasal immunization elicits an immune response at multiple mucosal sites, mice were immunized three times and local mucosal immune responses were analyzed by indirect ELISA (Figure 2A). On days 17 and 21, specific sIgA was successfully detected in fecal extract, bronchoalveolar lavage fluid, and enteral lavage fluid in all mice inoculated with CaP-VLPs (Figure 2B). All the experimental groups vaccinated with CaP-VLPs produced antibodies anti inactivated FMDV. The specific sIgA in the small intestine and stool mainly concentrated on day 17 after immunization and decreased on day 21, while the specific sIgA detected in alveolar lavage fluid could still maintain a high level on day 21.

### Humoral immune responses

**Figure 3:**
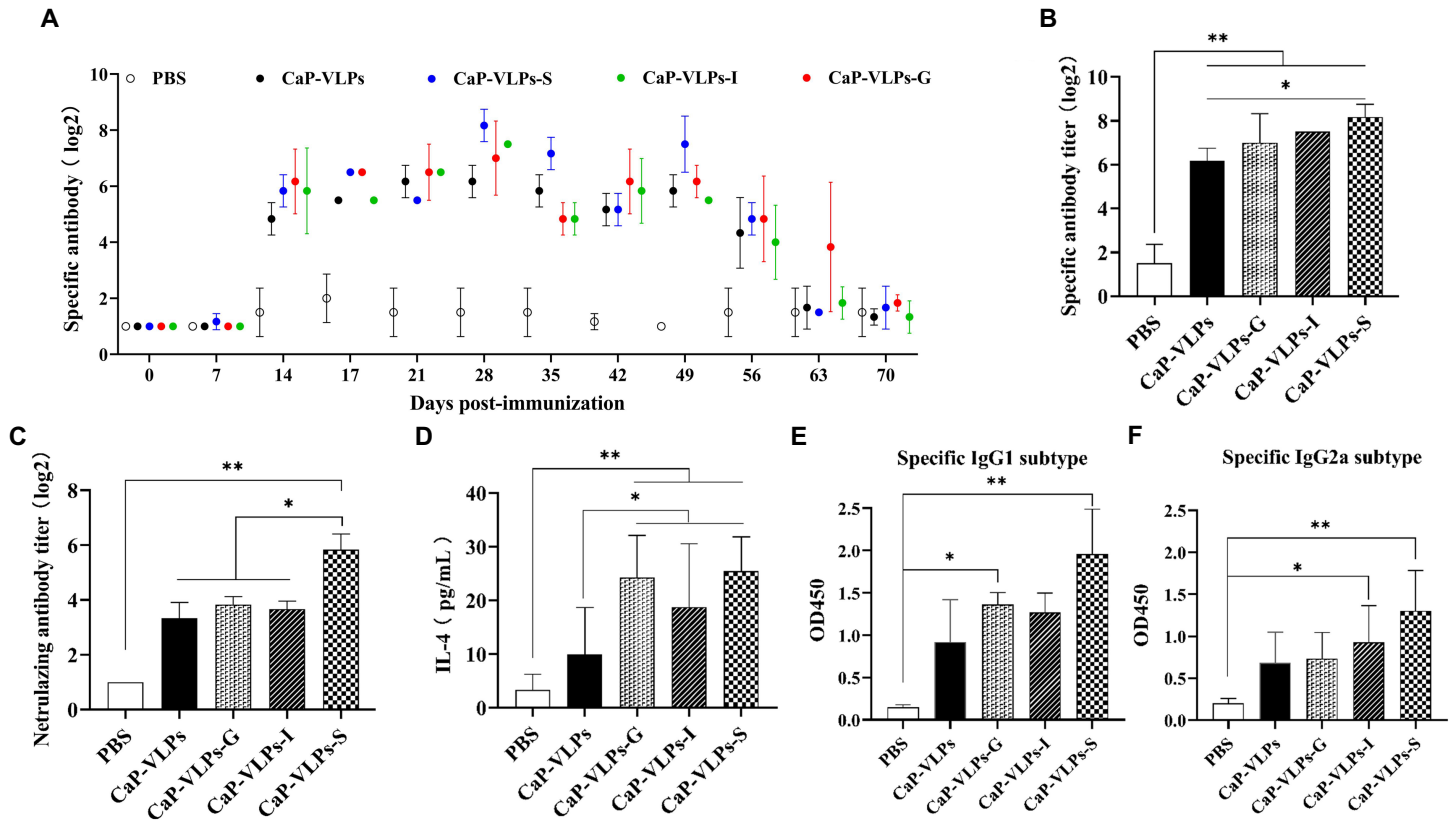
FMDV-specific humoral immune responses induced by CaP-VLPs in mice. **(A)** Changes in antibody titers against type-O FMDV were determined by LPBE assay after vaccination. **(B)** Titers of IgG of experimental groups on day 28 after the first immunization. **(C)** Titers of neutralizing antibodies of experimental groups on day 28 after the first immunization. **(D)** Content of IL-4 in the serum of mice on day 28 after the first immunization. **(E)** Content of the IgG1 subtype in serum on day 28 after the first immunization. **(F)** Content of the IgG2a subtype in serum on day 28 after the first immunization. Asterisks indicate significant differences (*p < 0.05, **p < 0.01).

Nasopharyngeal associated lymphoid tissue is a unique induction site for B cell response and plasma cell generation, allowing the intranasal vaccine administration to effectively induce systemic antibodies (Brandtzaeg, 2011). To evaluate the systemic humoral immune response elicited by intranasal vaccination, we measured FMDV-specific antibodies in mouse serum by LPBE. In natural hosts of FMD, such as cattle, sheep, and pigs, the serum antibody titer measured by LPBE is positively correlated with the results of animal challenge protection, LPBE is considered to be the gold standard for evaluating the immune effect of FMD vaccine (Lu et al., 2008; Ma et al., 2011). Anti-FMDV-specific serum IgG was produced in each immunization group, and the high level of IgG titer was maintained for up to 7 weeks. It should be noted that the serum of the mice already contained high levels of antibodies on day 14, suggesting that activated lymphocytes had been efficiently circulating *in vivo* after the first two intranasal immunizations (Figure 3A). In addition, serum IgG of most immune groups reached the highest level on day 28. The antibody titer of the CaP-VLPs-S group was significantly higher than that of the CaP-VLPs group, and there was no significant difference between the antibody titers of other adjuvant groups and the CaP-VLPs group (Figure 3B). Next, the neutralization effect of mouse serum antibodies against the specific strain of type-O FMDV was tested by virus neutralization assay. Figure 3C showed that the neutralizing antibody titers induced by the CaP-VLPs vaccine combined with S adjuvant were significantly higher than those induced by other experimental groups. The above results supported that the VLPs vaccine biomineralized by calcium phosphate can induce an effective humoral immune response after mucosal immunization. IL-4 is a Th2-type cytokine that can promote the proliferation and activation of B cells and differentiation into plasma cells, and is a key regulator of humoral and adaptive immune responses (Yokota et al., 1986). On day 28, mice immunized with CaP-VLPs in combination with adjuvant induced higher levels of serum IL-4 than CaP-VLPs alone (Figure 3D).

We further evaluated IgG subtype responses in mice immunized with CaP-VLPs with or without adjuvant to compare the strength of Th1 and Th2 responses. Levels of the IgG1 and IgG2a antibody isotypes reflect Th2 immunity and Th1 immunity, respectively. CaP-VLPs with adjuvant effectively increased the IgG1 and IgG2a responses (Figures 3E,F).

### Cellular immune responses

**Figure 4:**
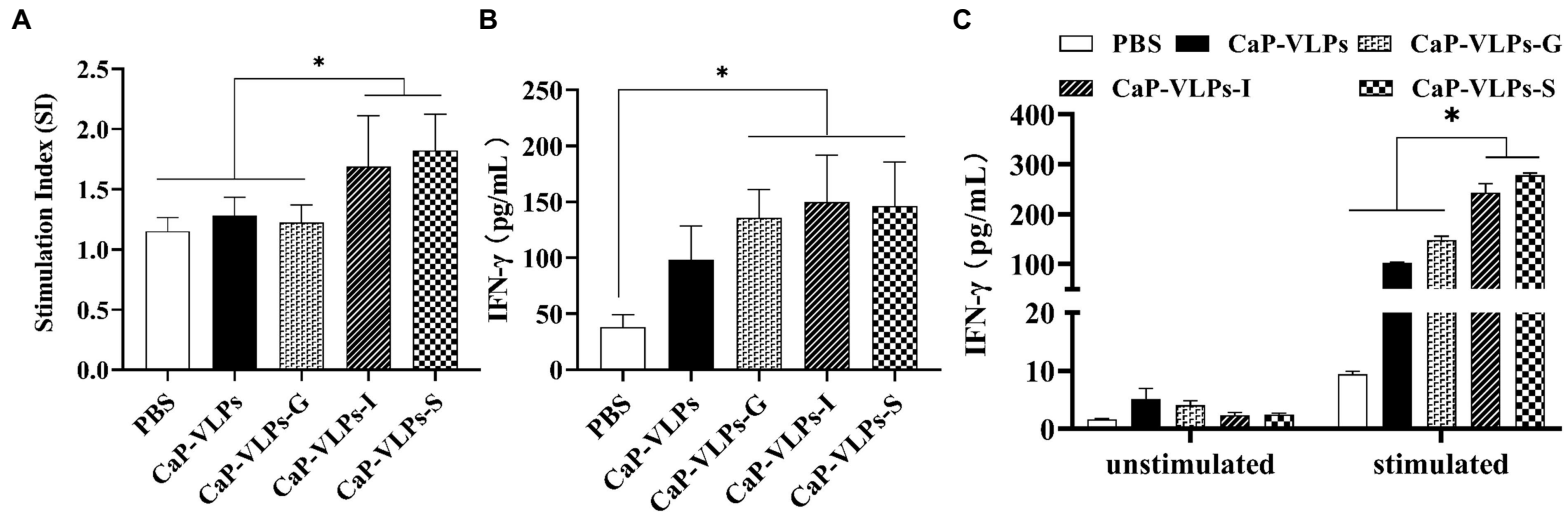
The cellular immune response induced by CaP-VLPs in mice. **(A)** Detection of T-lymphocyte proliferation on day 28 after the first immunization. **(B)** Content of IFN-γ in the serum of mice on day 28 after the first immunization. **(C)** Changes of IFN-γ content in spleen lymphocyte culture supernatant before and after antigen stimulation. Asterisks indicate a significant difference (*p < 0.05).

To determine whether intranasal inoculation of CaP-VLPs can induce a specific cellular immune response, spleen lymphocytes were collected on day 28 after the first dose and re-stimulated with the VLPs *ex-vivo*, followed by detection of cell proliferation to compare the intensity of cellular immune responses elicited by each immune group. The immunization group with S or I adjuvant effectively induced proliferation of lymphocytes under antigen stimulation (Figure 4A). IFN-γ is a marker cytokine of Th1-type cells and is considered a key regulator of cellular immune responses (Otani et al., 2021). Therefore, we detected the IFN-γ content in serum and the supernatant of spleen lymphocytes collected after antigen stimulation. The serum IFN-γ content in the immunized group was higher than that in the control group, indicating that CaP-VLPs can effectively induce the immune response. However, there was no significant difference between the experimental groups (Figure 4B). The stimulated culture supernatant of spleen lymphocytes contained a large amount of IFN-γ, further demonstrating that the CaP-VLPs could induce cellular immune responses through nasal immunization, and adjuvant enhanced the strength of this immune response (Figure 4C).

### Morphological observation of lungs

**Figure 5:**
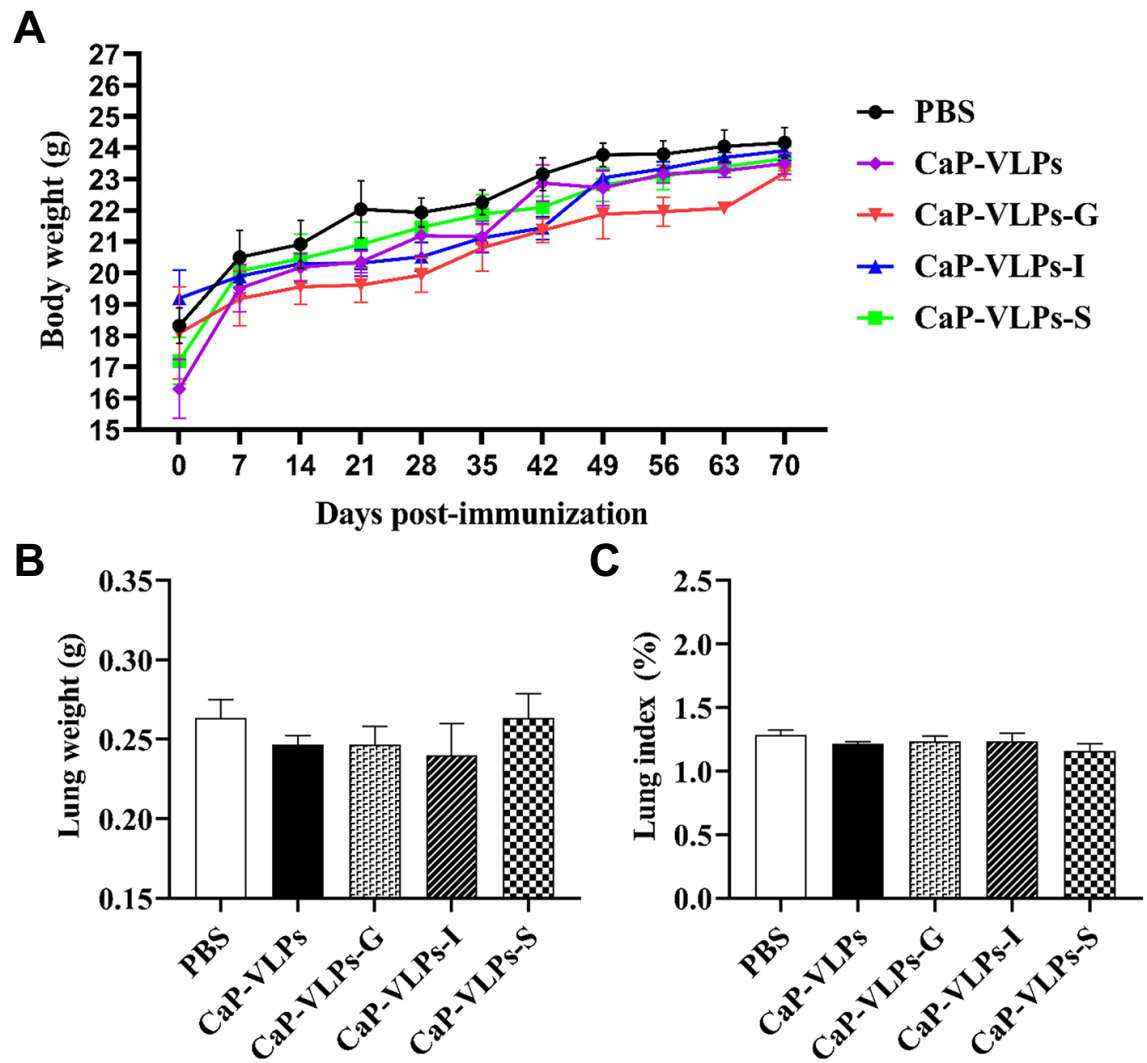
Changes in lung macroscopic parameters in mice on day 28 after the first immunization. **(A)** Weight change in mice. **(B)** The lung weight. **(C)** The lung index.

During the immunization period, the animals did not show any signs of discomfort or significant weight loss (Figure 5A). There was no significant difference in lung weight (Figure 5B) or organ index (Figure 5C) between the experimental and control group. Thus, the vaccine did not have a significant impact on the growth and development of mice after intranasal inoculation.

### Histopathological observation of lungs

**Figure 6:**
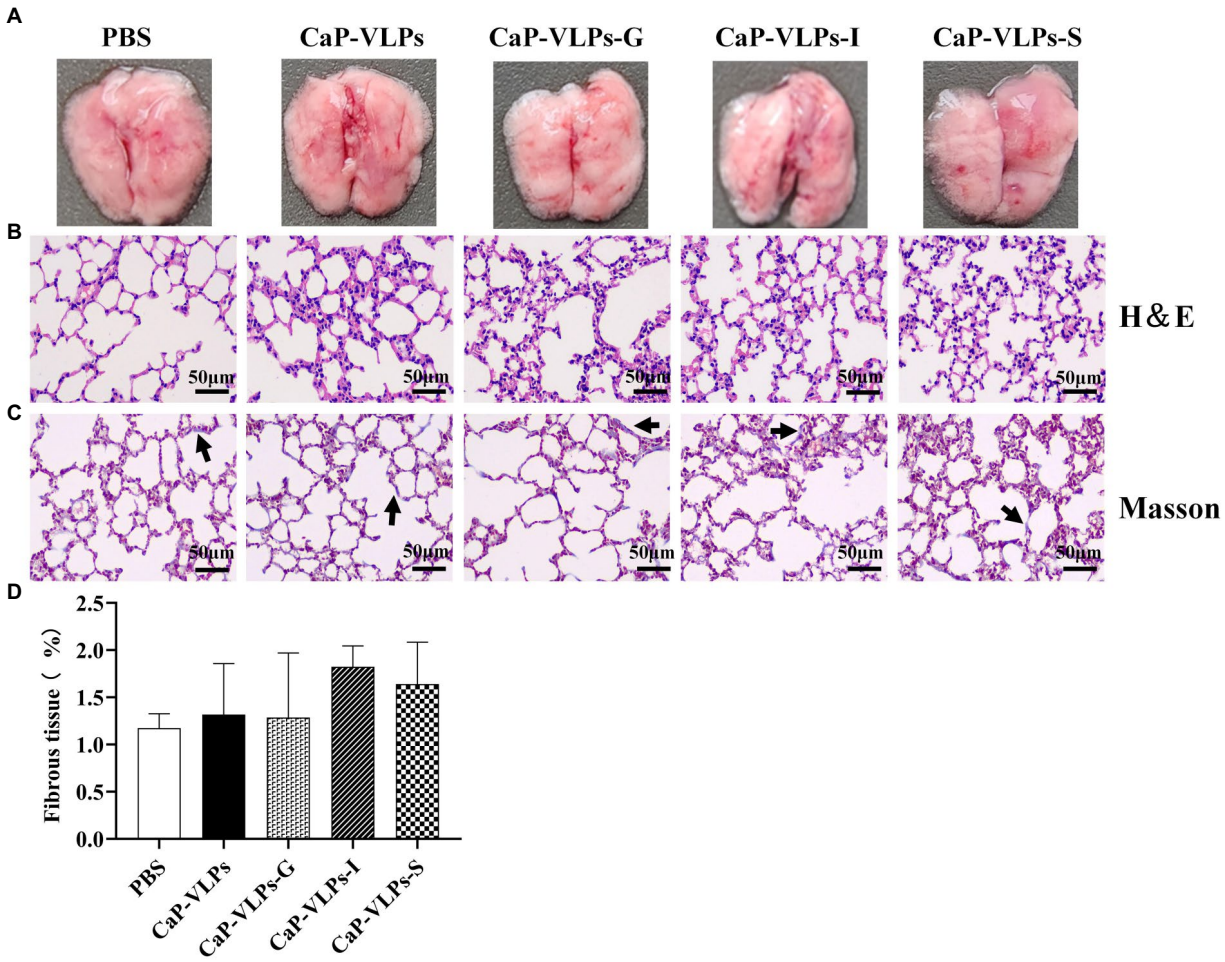
Gross observation and histopathological analysis of lungs. **(A)** The lung macroscopic structure on day 28 after the first immunization. **(B)** Representative photographs of H&E-stained lung sections in each group (black bar = 50 μm). **(C)** Representative micrographs of Masson trichrome staining, with blue-stained collagen fibers (black arrow; black bar = 50 μm). **(D)** Percentage of fibrous tissue expression area in lung tissue.

Gross observation revealed that the lungs of mice in each group were uniform in texture, rose-colored, elastic to touch, and showed no obvious signs of lesions (Figure 6A). H&E staining and histopathological score showed that the lungs in each group had normal and complete histological structure, with clean alveoli, alveolar ducts, and alveolar cavities. The morphology of alveolar epithelial cells was normal, no obvious degeneration or necrosis was observed, and no obvious inflammatory cell infiltration was observed in the interstitium (Figure 6B; Table 1). The connective tissue network of the lung is mainly composed of collagen fibers, which are mainly located in the loose connective tissue below the epithelium and oriented circularly or obliquely around the bronchiole. Using Masson trichrome staining to assess collagen fiber expression in lung tissue, no significant difference in the expression level of collagen fibers in the lung tissue of each group of animals was observed (Figures 6C,D).

**Table 1:** H&E score of inflammatory infiltration of lung tissue.

Immune group	Inflammation score
PBS	(−)
CaP-VLPs	(−)
CaP-VLPs-G	(−)
CaP-VLPs-I	(−)
CaP-VLPs-S	(−)

Using the 4-level method, the degree of lesions was recorded as mild (+), mild (++), moderate (+++), and severe (++++); if there was no lesion, it was recorded as (−).

## Discussion

Invading the host body through the respiratory tract and the digestive tract is the main route of transmission of the FMDV (Stenfeldt et al., 2016, 2018). A dose of only 8 infectious units (IU) can cause sheep infection and cause lesions (Sellers and Gloster, 2008). Vaccination can effectively control the spread of FMD and reduce the clinical symptoms caused by the disease, but cannot prevent primary infection of the nasopharyngeal mucosa (Stenfeldt et al., 2015). The immune response induced by the mucosal immune system can play a protective immune role in the initial stage of pathogen invasion, which is significant for disease prevention and control. Intranasal immunization is an ideal method of vaccination, and can provoke a wide range of mucosal and systemic immune responses (Isaka et al., 1998; Alpar et al., 2001).

In this study, we tested the immune responses elicited by FMD CaP-VLPs inoculated in mice *via* the nasal mucosal route in the presence or absence of adjuvants. It should be noted that the weak immunogenicity of subunit vaccines and the properties of mucosal sites often result in the inability of vaccines to induce an effective protective immune response, these recombinant vaccines require adjuvants to obtain protection similar to conventional vaccines (Hu et al., 2001; Feng et al., 2022; Kimoto, 2022). Hence, we screened several mucosal adjuvants to use with the CaP-VLPs vaccine.

We detected the presence of sIgA in the lungs, small intestines, and feces of vaccine-immunized mice, indicating that intranasal immunization of CaP-VLPs can effectively induce immune responses at multiple mucosal sites. Moreover, specific antibodies against FMDV were also produced in the serum of mice, and the titer of serum antibodies reached 1:360. The proportion of the IgG1 subtype in total serum was higher than that of the IgG2a subtype, revealing the immune response induced by intranasal immunization of CaP-VLPs was mainly humoral immunity. It is important to note that both LPBE and indirect ELISA have high confidence because they use inactivated FMDV rather than VLPs in the detection process, eliminating the potential interference of immunizing antigens as detection antigens. Virus neutralization experiments have shown that CaP-VLPs can induce the production of neutralizing antibodies, while the neutralizing antibodies of foot-and-mouth disease virus can effectively bind virions and inhibit the proliferation and transmission of the virus in the body, playing a key role in resisting viral infection (He et al., 2021). Analysis of spleen lymphocyte proliferation confirmed the existence of a large number of memory T cells in spleen cells, indicating that CaP-VLPs can be effectively absorbed by cells in the nasal mucosa, and the activated immune cells can settle down in the distal mucosa and peripheral lymphoid organs *via* lymphatic and blood circulation. These results above indicate that the core-shell nanomaterials have the potential for clinical application as intranasal vaccine preparation. This may be due to the ability of amorphous CaP nanoparticles to adhere to cells (Balasundaram et al., 2006), overcoming the mechanism of foreign body removal naturally present on the mucosal surface, which enhances the adsorption of vaccines at mucosal sites. Furthermore, by comparing the sIgA content in mucosal sites, serum-specific IgG titers, and other indicators, we found that the immune response induced by the S adjuvant group was significantly better than other immune groups, confirming that the S adjuvant can be used mucosal immunity to CaP-VLPs.

Biocompatibility is an important issue to consider when using an antigen carrier or adjuvant. To further confirm the biocompatibility of the mineralized complex, morphology and histopathology of the lungs were analyzed. Lungs with uniform pink color in all test groups exhibited normal macroscopic structure and no obvious change in volume, which is consistent with the lung mass and lung index shown in Figures 5B,C. Through histological observation and histopathological score, the lungs of each test group were shown to have a normal and complete histological structure. In summary, after immunization, there was no obvious pathological change in the lungs of each test group, CaP-VLPs vaccine has good biological safety.

## Conclusion

We used a novel nano vaccine with a core-shell structure for mucosal immunity for the first time. Our results showed that CaP-VLPs can be effectively taken up by epithelial cells in mucosal sites and induced antigen-specific systemic humoral and cellular immune responses. S adjuvant effectively enhanced the immune response induced by CaP-VLPs and provided better protection for animals. Considering FMD is mainly transmitted through the respiratory and digestive tracts, our study supports that CaP-VLPs nanocomposites adjuvanted with S can be used for clinical mucosal vaccination to better control FMD outbreaks.

## Data availability statement

The original contributions presented in the study are included in the article/Supplementary material, further inquiries can be directed to the corresponding authors.

## Ethics statement

The animal study was reviewed and approved by Animal Ethics Committee of Lanzhou Veterinary Research Institute, Chinese Academy of Agricultural Sciences.

## Author contributions

HG conceived the study. SY designed the experiments. SL interpreted the results and wrote the manuscript. SL, HS, RZ, and SP executed experiments and analyzed the data. YZ, SS, MB, and HD contributed reagents, materials, and analysis tools. All authors contributed to the article and approved the submitted version.

## Funding

This research was supported by grants from the Science and Technology Major Project of Gansu Province (21ZD3NA001), the National Key Research and Development Program (2021YFD1800300), the National Natural Science Foundation of China (32072847, 31873023, 32072859, and 32002272), and Science and Technology Talents and Platform Program (202205AF150007).

## Conflict of interest

The authors declare that the research was conducted in the absence of any commercial or financial relationships that could be construed as a potential conflict of interest.

## Publisher’s note

All claims expressed in this article are solely those of the authors and do not necessarily represent those of their affiliated organizations, or those of the publisher, the editors and the reviewers. Any product that may be evaluated in this article, or claim that may be made by its manufacturer, is not guaranteed or endorsed by the publisher.
