# Supplementary material for: Local and systemic immune responses induced by intranasal immunization with biomineralized foot-and-mouth disease virus-like particles
Source: Front Microbiol. 2023 Feb 3;14:1112641. doi: 10.3389/fmicb.2023.1112641 (PMC9937024; doi:10.3389/fmicb.2023.1112641)
Supplement: Supplementary file 1 [file Data_Sheet_1.doc]

**Local and systemic immune responses induced by intranasal immunization with biomineralized foot-and-mouth disease virus-like particles**

**Shuo Li^1, †^, Ruichong Zhao^1, †^, Hetao Song^1^, Songjia Pan^1^, Yun Zhang^1^, Hu Dong^1^, Manyuan Bai^1^, Shiqi Sun^1^, Huichen Guo^1,2,3, *^, Shuanghui Yin^1,*^**

^1^State Key Laboratory of Veterinary Etiological Biology, College of Veterinary Medicine, Lanzhou University, Lanzhou Veterinary Research Institute, Chinese Academy of Agricultural Sciences, Lanzhou, Gansu, China;

^2^College of Veterinary Medicine, Gansu Agricultural University, Lanzhou 730070, China；

^3^Yunnan Tropical and Subtropical Animal Virus Diseases Laboratory, Yunnan Animal Science and Veterinary Institute, Kunming, Yunnan, China

***Corresponding author:**

Shuanghui Yin

State Key Laboratory of Veterinary Etiological Biology, College of Veterinary Medicine, Lanzhou University, Lanzhou Veterinary Research Institute, Chinese Academy of Agricultural Sciences, Lanzhou, Gansu, 730000, China;

E-mail: yinshuanghui@caas.cn

Tel: +86-13669397146.

**Data Sheet:**

Supplemental Table 3. Immune responses of regional mucosal sites elicited by CaP-VLPs (n=3).

| Group | Days post first immunization | IgA, intestine (n=3) | | | IgA, lung (n=3) | | | IgA, feces (n=3) | | |
| --- | --- | --- | --- | --- | --- | --- | --- | --- | --- | --- |
|  |  | 1 | 2 | 3 | 1 | 2 | 3 | 1 | 2 | 3 |
| PBS | 17 | 0.337 | 0.286 | 0.272 | 0.156 | 0.144 | 0.134 | 0.33 | 0.2 | 0.272 |
|  | 21 | 0.254 | 0.231 | 0.233 | 0.137 | 0.139 | 0.138 | 0.212 | 0.212 | 0.215 |
|  | 28 | 0.227 | 0.311 | 0.32 | 0.137 | 0.157 | 0.11 | 0.467 | 0.231 | 0.23 |
|  | 35 | 0.245 | 0.221 | 0.178 | 0.105 | 0.102 | 0.107 | 0.184 | 0.176 | 0.131 |
| CaP-VLPs | 17 | 0.699 | 0.851 | 0.69 | 0.669 | 0.651 | 0.389 | 0.301 | 0.549 | 0.61 |
|  | 21 | 0.645 | 0.431 | 0.478 | 0.595 | 0.641 | 0.547 | 0.34 | 0.418 | 0.354 |
|  | 28 | 0.677 | 0.189 | 0.249 | 0.317 | 0.125 | 0.187 | 0.254 | 0.295 | 0.251 |
|  | 35 | 0.277 | 0.192 | 0.237 | 0.408 | 0.397 | 0.104 | 0.208 | 0.237 | 0.256 |
| CaP-VLPs-G | 17 | 1.001 | 0.781 | 0.647 | 0.486 | 0.47 | 0.647 | 0.431 | 0.776 | 0.648 |
|  | 21 | 0.525 | 0.672 | 0.587 | 0.472 | 0.616 | 0.751 | 0.498 | 0.486 | 0.471 |
|  | 28 | 0.224 | 0.335 | 0.308 | 0.183 | 0.21 | 0.41 | 0.248 | 0.217 | 0.28 |
|  | 35 | 0.229 | 0.302 | 0.234 | 0.185 | 0.152 | 0.153 | 0.212 | 0.296 | 0.309 |
| CaP-VLPs-I | 17 | 0.906 | 0.643 | 0.626 | 0.433 | 0.489 | 0.456 | 0.729 | 0.575 | 0.775 |
|  | 21 | 0.565 | 0.675 | 0.706 | 0.684 | 0.822 | 0.628 | 0.539 | 0.635 | 0.351 |
|  | 28 | 0.469 | 0.464 | 0.433 | 0.496 | 0.292 | 0.184 | 0.258 | 0.255 | 0.23 |
|  | 35 | 0.258 | 0.251 | 0.492 | 0.177 | 0.118 | 0.132 | 0.182 | 0.135 | 0.203 |
| CaP-VLPs-S | 17 | 1.319 | 1.126 | 0.843 | 0.669 | 0.897 | 0.476 | 0.797 | 1.294 | 1.028 |
|  | 21 | 0.523 | 0.935 | 0.582 | 0.509 | 0.743 | 0.769 | 0.636 | 0.748 | 0.754 |
|  | 28 | 0.433 | 0.236 | 0.341 | 0.521 | 0.149 | 0.168 | 0.362 | 0.244 | 0.281 |
|  | 35 | 0.251 | 0.315 | 0.606 | 0.176 | 0.318 | 0.15 | 0.507 | 0.26 | 0.319 |

Supplemental Table 2. FMDV-specific Humoral immune responses in mice (n=3).

| Days post first immunization | Specific antibodies from mice serum were measured by LPBE (log2) | | | | | | | | | | | | | | |
| --- | --- | --- | --- | --- | --- | --- | --- | --- | --- | --- | --- | --- | --- | --- | --- |
|  | PBS (n=3) | | | CaP-VLPs (n=3) | | | CaP-VLPs-G (n=3) | | | CaP-VLPs-I (n=3) | | | CaP-VLPs-S (n=3) | | |
|  | 1 | 2 | 3 | 1 | 2 | 3 | 1 | 2 | 3 | 1 | 2 | 3 | 1 | 2 | 3 |
| 0 | 1 | 1 | 1 | 1 | 1 | 1 | 1 | 1 | 1 | 1 | 1 | 1 | 1 | 1 | 1 |
| 7 | 1 | 1 | 1 | 1 | 1 | 1 | 1 | 1.5 | 1 | 1 | 1 | 1 | 1 | 1 | 1 |
| 14 | 1 | 2.5 | 1 | 5.5 | 4.5 | 4.5 | 5.5 | 5.5 | 6.5 | 5.5 | 7.5 | 5.5 | 4.5 | 5.5 | 7.5 |
| 17 | 1 | 2.5 | 2.5 | 5.5 | 5.5 | 5.5 | 6.5 | 6.5 | 6.5 | 6.5 | 6.5 | 6.5 | 5.5 | 5.5 | 5.5 |
| 21 | 1 | 2.5 | 1 | 5.5 | 6.5 | 6.5 | 5.5 | 5.5 | 5.5 | 6.5 | 5.5 | 7.5 | 6.5 | 6.5 | 6.5 |
| 28 | 1 | 2.5 | 1 | 5.5 | 6.5 | 6.5 | 8.5 | 8.5 | 7.5 | 5.5 | 7.5 | 8 | 7.5 | 7.5 | 7.5 |
| 35 | 1 | 2.5 | 1 | 5.5 | 6.5 | 5.5 | 7.5 | 6.5 | 7.5 | 4.5 | 4.5 | 5.5 | 4.5 | 4.5 | 5.5 |
| 42 | 1.5 | 1 | 1 | 5.5 | 5.5 | 4.5 | 4.5 | 5.5 | 5.5 | 5.5 | 5.5 | 7.5 | 4.5 | 6.5 | 6.5 |
| 49 | 1 | 1 | 1 | 5.5 | 5.5 | 6.5 | 6.5 | 7.5 | 8.5 | 5.5 | 6.5 | 6.5 | 5.5 | 5.5 | 5.5 |
| 56 | 2.5 | 1 | 1 | 3 | 5.5 | 4.5 | 4.5 | 4.5 | 5.5 | 6.5 | 4.5 | 3.5 | 5.5 | 3.5 | 3 |
| 63 | 2.5 | 1 | 1 | 2.5 | 1 | 1.5 | 1.5 | 1.5 | 1.5 | 2.5 | 6.5 | 2.5 | 2.5 | 1.5 | 1.5 |
| 70 | 2.5 | 1 | 1 | 1 | 1.5 | 1.5 | 1 | 1.5 | 2.5 | 2 | 1.5 | 2 | 2 | 1 | 1 |

Supplemental Table 3. Neutralizing antibody titer from mouse serum at 28 days post-first immunization (n=3).

| Group (vaccine) | Mouse No. | Neutralizing antibody titer (log2) |
| --- | --- | --- |
| PBS | 1 | 1 |
|  | 2 | 2.5 |
|  | 3 | 1 |
| CaP-VLPs | 1 | 5.5 |
|  | 2 | 6.5 |
|  | 3 | 6.5 |
| CaP-VLPs-G | 1 | 5.5 |
|  | 2 | 7.5 |
|  | 3 | 8 |
| CaP-VLPs-I | 1 | 7.5 |
|  | 2 | 7.5 |
|  | 3 | 7.5 |
| CaP-VLPs-S | 1 | 8.5 |
|  | 2 | 8.5 |
|  | 3 | 7.5 |

Supplemental Table 4. Antigen-specific IgG2a and IgG1 level from mouse serum at 28 days post-first immunization (n=3).

| Group (vaccine) | Mouse No. | IgG1 antibody | IgG2a antibody |
| --- | --- | --- | --- |
| PBS | 1 | 0.18 | 0.261 |
|  | 2 | 0.1395 | 0.189 |
|  | 3 | 0.13 | 0.153 |
| CaP-VLPs | 1 | 0.4615 | 0.3565 |
|  | 2 | 0.844 | 0.6185 |
|  | 3 | 1.452 | 1.0735 |
| CaP-VLPs-G | 1 | 1.293 | 0.7 |
|  | 2 | 1.274 | 1.432 |
|  | 3 | 1.523 | 0.6435 |
| CaP-VLPs-I | 1 | 1.4545 | 0.3865 |
|  | 2 | 1.017 | 0.9895 |
|  | 3 | 1.34 | 0.8215 |
| CaP-VLPs-S | 1 | 2.201 | 1.8455 |
|  | 2 | 1.354 | 1.1275 |
|  | 3 | 2.325 | 0.9295 |

Supplemental Table 5. The concentrations (pg/ml) of IFN-γ and IL-4 in the mouse serum (n=3) were measured by ELISA 28 days after the first nasal immunization.

| Group | Mouse No. | IL-4 concentration (serum, pg/ml) | IFN-γ concentration (serum, pg/ml) |
| --- | --- | --- | --- |
| PBS | 1 | 0.825 | 48.421 |
|  | 2 | 2.624 | 26.577 |
|  | 3 | 6.515 | 39.690 |
| CaP-VLPs | 1 | 2.477 | 125.589 |
|  | 2 | 7.872 | 103.225 |
|  | 3 | 19.547 | 66.382 |
| CaP-VLPs-G | 1 | 22.563 | 143.069 |
|  | 2 | 17.507 | 156.263 |
|  | 3 | 32.802 | 107.533 |
| CaP-VLPs-I | 1 | 32.323 | 159.204 |
|  | 2 | 12.803 | 185.817 |
|  | 3 | 11.190 | 103.943 |
| CaP-VLPs-S | 1 | 22.563 | 160.676 |
|  | 2 | 32.802 | 176.178 |
|  | 3 | 21.062 | 101.075 |

Supplemental Table 6. The detection of T-lymphocyte proliferation on day 28 after the first immunization and changes of IFN-γ content in lymphocyte culture supernatant before and after antigen stimulation.

| Group | Mouse No. | lymphocyte proliferation index | IFN-γ content  (unstimulated, pg/ml) | IFN-γ content  (stimulated, pg/ml) |
| --- | --- | --- | --- | --- |
| PBS | 1 | 1.03 | 1.81 | 9 |
|  | 2 | 1.259 | 1.73 | 9.4 |
|  | 3 | 1.167 | 1.49 | 9.95 |
| CaP-VLPs | 1 | 1.109 | 3.08 | 102.695 |
|  | 2 | 1.373 | 6.56 | 104.195 |
|  | 3 | 1.368 | 5.77 | 101.945 |
| CaP-VLPs-G | 1 | 2.175 | 3.48 | 154.622 |
|  | 2 | 1.46 | 4.98 | 139.793 |
|  | 3 | 1.427 | 3.95 | 150.439 |
| CaP-VLPs-I | 1 | 1.109 | 2.21 | 236.222 |
|  | 2 | 1.373 | 2.05 | 229.460 |
|  | 3 | 1.368 | 2.92 | 263.705 |
| CaP-VLPs-S | 1 | 2.15 | 2.29 | 282.427 |
|  | 2 | 1.76 | 2.45 | 273.318 |
|  | 3 | 1.561 | 2.77 | 278.598 |

Supplemental Table 7. The lung weight and the lung index.

| Group (vaccine) | Mouse No. | Lung weight (g) | Lung index (%) |
| --- | --- | --- | --- |
| PBS | 1 | 0.27 | 1.29 |
|  | 2 | 0.25 | 1.24 |
|  | 3 | 0.27 | 1.32 |
| CaP-VLPs | 1 | 0.24 | 1.22 |
|  | 2 | 0.25 | 1.23 |
|  | 3 | 0.25 | 1.2 |
| CaP-VLPs-G | 1 | 0.24 | 1.2 |
|  | 2 | 0.24 | 1.23 |
|  | 3 | 0.26 | 1.28 |
| CaP-VLPs-I | 1 | 0.22 | 1.17 |
|  | 2 | 0.24 | 1.23 |
|  | 3 | 0.26 | 1.3 |
| CaP-VLPs-S | 1 | 0.25 | 1.1 |
|  | 2 | 0.26 | 1.17 |
|  | 3 | 0.28 | 1.21 |

Supplemental Table 8. The percentage of fibrous tissue expression area in lung tissue

| Group (vaccine) | Mouse No. | Fibrous tissue （%） |
| --- | --- | --- |
| PBS | 1 | 1.23 |
|  | 2 | 1.29 |
|  | 3 | 1 |
| CaP-VLPs | 1 | 1.93 |
|  | 2 | 1.11 |
|  | 3 | 0.91 |
| CaP-VLPs-G | 1 | 1.79 |
|  | 2 | 1.62 |
|  | 3 | 2.06 |
| CaP-VLPs-I | 1 | 0.52 |
|  | 2 | 1.51 |
|  | 3 | 1.83 |
| CaP-VLPs-S | 1 | 1.29 |
|  | 2 | 2.14 |
|  | 3 | 1.49 |
